# Tritrophic Interactions Mediated by Zoophytophagous Predator-Induced Host Plant Volatiles

**DOI:** 10.1007/s10886-024-01501-1

**Published:** 2024-05-09

**Authors:** Bashiru Adams, Abdullahi Ahmed Yusuf, Baldwyn Torto, Fathiya Mbarak Khamis

**Affiliations:** 1 International Centre of Insect Physiology and Ecology (icipe), P.O. Box 30772-00100, Nairobi, Kenya; 2 Department of Zoology and Entomology, University of Pretoria, Private Bag X20, Hatfield, 0028 South Africa

**Keywords:** Tomato, Terpenes, Green leaf volatiles, Olfaction, Attractants, Repellents

## Abstract

The zoophytophagous mirid predator *Nesidiocoris tenuis* and the ectoparasitoid *Stenomesius japonicus* are important biological control agents for several agricultural pests including the invasive leafminer, *Phthorimaea absoluta*, a destructive pest of Solanaceous crops especially tomato in sub-Saharan Africa. However, little is known about how feeding by *N. tenuis* can influence the tritrophic interactions in the tomato plant. Here, we tested the hypothesis that *N. tenuis* phytophagy would influence the tritrophic olfactory interactions between the host plant tomato and pest, predator, and parasitoid. In olfactometer assays, *P. absoluta* females and *N. tenuis* adults were both attracted to constitutive volatiles released by the tomato plant. Whereas females of *P. absoluta* avoided volatiles released by *N. tenuis*-infested plants, *S. japonicus* females and *N. tenuis* adults were attracted to the induced volatiles. In coupled gas chromatography-electroantennographic detection (GC-EAD) recordings of intact and *N. tenuis*-infested plant volatiles, antennae of *P. absoluta* and *S. japonicus* females both detected eight components, whereas *N. tenuis* adults detected seven components which were identified by GC-mass spectrometry (GC-MS) as terpenes and green leaf volatiles (GLVs). Dose-response olfactometer bioassays revealed that the responses of *P. absoluta*, *N. tenuis*, and *S. japonicus* varied with the composition and concentration of blends and individual compounds tested from *N tenuis*-induced volatiles. Females of *P. absoluta* showed no preference for an eight-component blend formulated from the individual repellents including hexanal, (*Z*)-3-hexenyl butanoate, and δ-elemene identified in the volatiles. On the other hand, *S. japonicus* females were attracted to an eight-component blend including the attractants (*E*)-2-hexenal, (*Z*)-3-hexenol, methyl salicylate, β-phellandrene, and (*E*)-caryophyllene. Likewise, *N. tenuis* adults were attracted to a seven-component blend including the attractants β-phellandrene, δ-elemene, and (*E*)-caryophyllene identified in the volatiles. Our findings suggest that there is potential for the use of terpenes and GLVs to manage the insects in the tritrophic interaction.

## Introduction

Plants respond to herbivory through various mechanisms including the release of defense chemicals (Aratani et al. 2023; Fiaboe et al. 2023; Sugimoto et al. 2023) which may either act directly on herbivores or indirectly by attracting natural enemies (predators or parasitoids) to protect the plant against further herbivory (Liu et al. 2021; Ayelo et al., 2021; Abdollahipour et al. 2020; Gebreziher and Gebreziher 2020; Helms et al. 2019; Dicke and Baldwin 2010). Research has shown that herbivore-induced chemicals can be exploited to manage crop pests (Pérez-Hedo et al. 2015a, 2018, 2021).

The generalist mirid predator *Nesidiocoris tenuis* (Reuter) (Hemiptera: Miridae), native to the tropics (Sanchez et al. 2014) is used as a biological control agent for key agricultural pests of economic importance such as thrips (*Frankliniella occidentalis*), aphids (*Myzus persicae*), spider mites (*Tetranychus urticae*), whiteflies (*Bemisia tabaci* and *Trialeurodes vaporariorum*), and the invasive tomato leafminer, *Phthorimaea absoluta* Meyrick (Lepidoptera: Gelechiidae) (Bouagga et al. 2018; Pérez-Hedo et al. 2015a; Sanchez et al. 2014). Likewise, the ectoparasitoid *Stenomesius japonicus* (Ashmead) (Hymenoptera: Eulophidae), is an efficient native larval parasitoid of *P. absoluta*, with a parasitism rate of up to 45% under greenhouse tomato production (Sambo et al. 2022).

Since the mirid predator *N. tenuis* feeds on tomato in the absence of its prey *P. absoluta*, it is important to examine how phytophagy in the predator may influence the tomato plant-*P. absoluta-N.tenuis/S. japonicus* tritrophic interaction. Previous studies and our recent work revealed that terpenes from the tomato host- and non-host plants influence the host finding behaviour of *P. absoluta* and *N. tenuis* (Adams et al. 2023; Pasquale et al. 2023; Pérez-Hedo et al. 2018). Moreover, terpenes are known to contribute to plant defense against several insects of agricultural importance (Ayelo et al. 2022, 2021; Subramani et al. 2021; Gebreziher and Gebreziher 2020).

Here, we tested the hypothesis that *N. tenuis* phytophagy would influence the tritrophic interaction between the host plant tomato and pest, predator, and parasitoid. To achieve this, we investigated the olfactory responses of *P. absoluta*, *S. japonicus*, and *N. tenuis* to intact and *N. tenuis*-infested tomato plants. Next, we used coupled gas chromatography (GC)- electroantennographic detection (GC-EAD) and GC-mass spectrometry to identify EAD-active components from intact and *N. tenuis*-induced tomato volatiles. Finally, we used olfactometer assays to identify the behaviorally-active components and blends.

## Materials and Methods

### Plants

Tomato (*Solanum lycopersicum* L. cv. Moneymaker) seeds were obtained from Simlaw Seeds Company Ltd., Nairobi, Kenya. The tomato plants used for the study were grown in a screen house at the International Centre of Insect Physiology and Ecology (*icipe*), Duduville Campus, Nairobi, Kenya (1580 m, S ’1°13.243’ E 0’6º53.732’) under the same conditions and procedure as previously described (Adams et al. 2023). The plants used for the experiment were all in the vegetative stage (5–6 weeks old).

### Insects

All the insect species used in this study were reared in a laboratory (28 ± 2 °C, 50–60% RH, and 12:12 L: D light/dark photoperiod) at *icipe* on potted tomato plants (Cal J variety).

*Nesidiocoris tenuis* adults were collected from a farmer’s tomato field in Mwea (S 0° 36′ 31.3″ E 037° 22′ 29.7″) and were identified using molecular tools in our previous study (Adams et al. 2023). The mirid predator was reared on 4–8 weeks old tomato plants in Plexiglass cages (40 cm × 50 cm × 60 cm). They were fed on non-viable eggs (10 g/cage/week) of *Ephestia kuehniella* Zeuler and *Artemia* sp. (Koppert biological system, Veilingweg, Netherlands) and supplemented with 80% honey solution. Adult *N. tenuis* (1:1 sex ratio) 2–7 days old were used for the experiment.

*Phthorimaea absoluta* was reared on 6–8 weeks-old tomato plants in Plexiglass cages (40 cm × 50 cm × 60 cm). The moths were fed on 80% honey solution ad libitum. The colony was established from *P. absoluta-*infested tomato plants collected from a farmer’s field in Mwea (S 0° 36′ 31.3″ E 037° 22′ 29.7″) and was rejuvenated with fresh infested tomato leaves every three months to reduce inbreeding. Because in our rearing facility, adult females mate by day 3, we assumed that emerged adult females (2–3 days old) found in the cages with adult males of similar age, had mated and were gravid. As such, females (2–3 days old) were used for the experiment.

*Stenomesius japonicus* adults were obtained from the Animal Rearing and Containment Unit (ARCU) at *icipe*. The parasitoid was reared on 4–5 weeks-old tomato plants that had been previously exposed to *P. absoluta* adults for 2 days. After egg hatching (3–5 days post infestation), tomato plants containing first and second instars of *P. absoluta* were then exposed to adults of *S. japonicus* for oviposition in Perspex cages (20 cm × 20 cm × 30 cm) for 72 h. The tomato plants containing *S. japonicus*-parasitised larvae of *P. absoluta* were incubated in cages with the addition of fresh tomato leaves until parasitoid emergence. Emerged adults of *S. japonicus* were fed on 80% honey solution ad libitum. Gravid naïve females of *S. japonicus* (1–2 days old) were used for the experiment.

### Olfactometer Assays

The olfactory responses of *P. absoluta*, *S. japonicus*, and *N. tenuis* to tomato headspace odours from intact and *N. tenuis*-infested plants were tested in a Y-tube olfactometer (arm = 10 cm, stem = 14 cm, diameter = 3 cm). The infested tomato plants were obtained by exposing 4–5 weeks old tomato plants in Plexiglass cages (30 cm × 30 cm × 40 cm) (one plant per cage) to 20 *N. tenuis* adults (1:1 sex ratio) per cage for two days and seven days to obtain herbivore-induced volatiles after 2-days post-infestation (2-NDPI) and 7-days post-infestation (7-NDPI), respectively. These days were selected to capture adult and oviposition-induced (2-NDPI) and both the adult and nymph-induced (7-NDPI) host plant volatiles. Thus, 2-NDPI represents herbivore- and oviposition-induced plants, while 7-NDPI represents plants induced solely by herbivore feeding. In each bioassay, the pot containing a tomato plant was wrapped with aluminium foil to ensure that only headspace odours from the tomato plants were released into the Y-tube olfactometer arms. The potted tomato plants were then transferred into roasting bags (50 cm × 60 cm) (Lifetime Brands Europe Ltd, Vale Pits Road, Birmingham) (sterilized for 12 h in an oven at 100 °C) connected to the two arms of the Y-tube olfactometer. Charcoal-purified and humidified air was passed through a Teflon tube into the roasting bags containing the test plants to push the emitted odours into the arms of the Y-tube olfactometer, each at a flow rate of 350 mL/min. Air was then sucked out of the Y-tube olfactometer (flow rate of 700 mL/min) with an electric-powered air-free vacuum pump (Model: DAA-V174-EB, GAST manufacturing company, Benton Harbor, Michigan, United States). After optimization, we found that placing the olfactometer in a vertical position worked for all the insects. Hence, all the bioassays were conducted with the olfactometer placed in this position. The responses of females of *P. absoluta*, *S. japonicus*, and both sexes of *N. tenuis* adults to odour sources from (i) intact tomato plants and (ii) *N. tenuis*-infested tomato plants paired against charcoal-purified air (control) in dual choice combinations (Table 1) were tested in the Y-tube olfactometer. A night condition was simulated by placing a red fluorescent bulb about 2 m above the olfactometer which emitted about 1000 lx illuminations of red light. The bioassays were conducted from 6: 00 AM – 9: 00 AM and 5: 00 PM – 7: 00 PM (local time) for the moth *P. absoluta* and the parasitoid *S. japonicus* when they were actively searching for a host. For the predator *N. tenuis*, the bioassays were conducted during their prey searching period, from 8: 00 AM to 4: 00 PM (local time). Adults of the predator were starved for 3 h and all insects were removed from the infested plants before each experiment. In each of the choice bioassays, except for *N. tenuis*, 60 insects were tested individually, and each insect was given 10 min to make a choice. Only 30 *N. tenuis* adult sexes each were individually tested in each pairwise comparison due to the limited numbers of the mirid available to work with during the study. In the Y-tube olfactometer, any insect that walked past 5 cm of any arm of the Y-tube was considered to have made a choice. In each assay, six different plants were used (10 insects per plant). After every five insects were tested, the Y-tube was cleaned with acetone and the two arms were then switched to account for positional bias. No insect was re-used in the experiment. All the bioassays were conducted under controlled laboratory conditions (27 °C and 70% RH).

**Table 1:** Pairwise comparisons of odor sources tested in the Y-tube olfactometer assays

Experiments	Pairwise comparison of odor sources
A	Charcoal-purified air vs. Charcoal-purified air
B	Charcoal-purified air vs. Intact tomato plant
C	Charcoal-purified air vs. *N. tenuis*-infested tomato plant (2-NDPI)
D	Charcoal-purified air vs. *N. tenuis*-infested tomato plant (7-NDPI)
E	Intact tomato plant vs. *N. tenuis*-infested tomato plant (2-NDPI)
F	Intact tomato plant vs. *N. tenuis*-infested tomato plant (7-NDPI)
G	*N. tenuis*-infested tomato plant (2-NDPI) vs. *N. tenuis*-infested tomato plant (7-NDPI)

Where 2- and 7-NDPI = *Nesidiocoris tenuis*-infested tomato plants after 2- and 7-days post-infestation, respectively

### Collection and Analysis of Volatiles

Headspace odours were collected from intact and *N. tenuis*-infested tomato plants. Odors from ten replicates of each tomato plant group were collected using the push-pull system as previously described (Adams et al. 2023). Charcoal-purified air was passed through a glass tube containing distilled water to humidify the air into the odour source at a flow rate of 400 mL/min. Volatiles were then pulled (350 mL/min) from the odour source onto a precleaned Super-Q adsorbent trap (30 mg) (Analytical Research System, Gainesville, FL) for 24 h. The trapped odours were eluted with 150 µL gas chromatography (GC) grade dichloromethane (Analytical grade, Sigma-Aldrich, St, Louis, MO) under a stream of nitrogen gas. The samples were stored at -80 °C until analysis.

One microlitre of each sample was analyzed on an HP 7890 A series gas chromatography (GC) coupled with an HP 5975 C mass spectrometer (MS) (Agilent Technologies, Wilmington, USA) using 30 m × 0.25 mm i.d., 0.25 μm Agilent HP-5 MS capillary column. Analysis was done in the splitless mode with an oven temperature programme starting at 35 °C for 5 min, and then increased to a final temperature of 280 °C at 10 °C/ min and held for 10.5 min. Helium was used as the carrier gas at a flow rate of 1.0 mL/min. The GC/MS library (Adams2 1995; National Institute of Standards and Technology (NIST, 2008)) was used to tentatively identify the compounds by comparing their mass spectra, retention times (RTs), electron ionization spectrum, and Kovats retention indices (RIs). The identities of the compounds were confirmed using authentic standards available. The identified compounds were quantified (ng/plant/h) using calibration curves (peak area vs. concentration) generated by serial dilutions of the authentic standards hexanal, (*Z*)-3-hexenol, (*Z*)-3-hexenyl acetate, α-pinene, methyl salicylate, and (*E*)-caryophyllene. The authentic standards were analyzed under the same GC/MS conditions outlined earlier at five different concentrations (1, 10, 100, 250, and 500 ng/µL). A mixture of n-alkane standards (C_8_–C_31_) was used to determine the RIs and were compared with online literature values.

**Table 2:** Mean amount (ng plant^− 1^ h^− 1^) of the released volatiles in the headspace odors of intact and *Nesidiocoris tenuis*-infested tomato plants (*N* = 10)

Peak no.	*R*.T (min)	*R*.I (Calc)	*R*.I (Lit)	Compound	Mean amount detected (ng plant^− 1^h^− 1^) ± SEM			*P*-value
					Intact	*Nesidiocoris tenuis*-infested		
						2-NDPI	7-NDPI	
1	6.31	783	786 ^A^	Hexanal ^ϕ^	2.68 ± 1.44 ^b^	8.98 ± 5.22 ^ab^	30.39 ± 8.73 ^a^	**0.012**
2	7.79	842	844 ^A^	(*E*)-2-Hexenal ^ϕ^	-	0.11 ± 0.08 ^b^	3.25 ± 0.28 ^a^	**0.034**
3	8.89	850	848 ^A^	(*Z*)-3-Hexenol ^ϕ^	-	1.59 ± 0.27	3.14 ± 0.26	0.942
4	9.46	903	906 ^B^	α-Thujene	0.11 ± 0.02	0.15 ± 0.06	0.21 ± 0.04	0.182
5	9.67	912	912 ^B^	α-Pinene ^ϕ^	14.62 ± 5.60	33.19 ± 14.70	35.11 ± 7.86	0.126
6	9.81	926	928 ^C^	Camphene ^ϕ^	1.53 ± 0.68	1.19 ± 0.24	0.82 ± 0.30	0.52
7	10.48	949	951 ^D^	*o*-Cymene ^ϕ^	15.18 ± 2.92 ^b^	15.67 ± 5.52 ^b^	31.52 ± 3.50 ^a^	**0.007**
8	10.59	959	960 ^A^	β-Pinene ^ϕ^	0.29 ± 0.15 ^b^	0.41 ± 0.18 ^ab^	3.77 ± 2.34 ^a^	**0.022**
9	10.63	964	961 ^D^	(*E*)-Isolimonene	-	3.89 ± 0.45 ^a^	0.58 ± 0.12 ^b^	**0.001**
10	10.91	971	973 ^B^	β-Myrcene ^ϕ^	1.72 ± 0.87	2.03 ± 1.24	2.94 ± 1.01	0.176
11	11.08	976	979 ^D^	δ-2-Carene ^ϕ^	180.26 ± 38.16 ^b^	229.15 ± 76.89 ^ab^	315.45 ± 33.05 ^a^	**0.013**
12	11.15	983	985 ^A^	α-Phellandrene ^ϕ^	66.81 ± 13.52	63.52 ± 18.82	85.58 ± 10.61	0.161
13	11.27	987	986 ^E^	δ-3-Carene ^ϕ^	0.28 ± 0.10	0.29 ± 0.14	0.32 ± 0.07	0.346
14	11.41	991	992 ^D^	α-Terpinene ^ϕ^	21.07 ± 5.59	19.57 ± 6.67	26.79 ± 3.63	0.242
15	11.51	993	992 ^F^	(*Z*)-3-Hexenyl acetate ^ϕ^	-	2.67 ± 0.27 ^b^	4.07 ± 0.18 ^a^	**< 0.001**
16	11.55	999	1000 ^D^	*p*-Cymene ^ϕ^	0.29 ± 0.12 ^ab^	0.07 ± 0.03 ^b^	0.51 ± 0.21 ^a^	**0.048**
17	11.63	1001	1005 ^D^	β-Phellandrene ^ϕ^	892.36 ± 166.31	703.46 ± 94.92	902.93 ± 97.24	0.595
18	12.18	1042	1042 ^A^	γ-Terpinene ^ϕ^	0.07 ± 0.05 ^b^	4.34 ± 2.36 ^a^	6.23 ± 2.45 ^a^	**0.002**
19	14.27	1174	1179 ^G^	(*Z*)-3-Hexenyl butanoate ^ϕ^	-	3.42 ± 0.22 ^b^	10.30 ± 0.25 ^a^	**< 0.001**
20	14.47	1199	1200 ^G^	Methyl salicylate ^ϕ^	-	5.10 ± 0.25	9.46 ± 0.35	0.411
21	16.58	1313	1315 ^D^	δ-Elemene ^ϕ^	2.97 ± 2.15 ^b^	2.99 ± 2.67 ^b^	10.36 ± 2.74 ^a^	**0.001**
22	17.23	1386	1391 ^A^	α-Cedrene ^ϕ^	-	-	0.17	
23	17.72	1389	1396 ^A^	(*E*)-Caryophyllene ^ϕ^	13.90 ± 4.59 ^b^	36.36 ± 11.09 ^ab^	72.01 ± 10.67 ^a^	**< 0.001**
24	18.17	1428	1430 ^A^	α-Humulene ^ϕ^	0.35 ± 0.21 ^b^	3.72 ± 2.24 ^ab^	10.58 ± 3.11 ^a^	**< 0.001**
25	18.78	1438	1441 ^A^	α-Guaiene	-	1.21 ± 0.22	1.85 ± 0.32	0.53
26	19.74	1559	1552 ^H^	Cadina-1,4-diene	-	-	2.32 ± 0.42	

R.T (min) = Retention time in minutes, R.I (Calc) = Retention index calculated based on the C8- C31 n- alkanes of HP- 5 MS column, R.I (Lit) = Retention index obtained from literature: A = (Njuguna et al. 2018), B = (Antwi-Agyakwa et al. 2021), C = (Pavlović et al. 2006), D = (Kihika et al. 2020), E = (Fiaboe et al. 2023), F = (Jain et al. 2001), G = (Ayelo et al., 2021), H = (Jalali-Heravi et al. 2006). P-value = Probability value of the non-parametric test for comparing the amounts of volatiles released by the intact and *N. tenuis*-infested tomato plants where significant values are in bold and mean with different letters are significantly different at 5% probability level (*P* < 0.05), the symbols (-) = not detected, (^ϕ^) = compounds confirmed with authentic standards. The acronyms 2- and 7-NDPI = *Nesidiocoris tenuis*-infested tomato plants after 2- and 7-days post-infestation, respectively

### Electrophysiology

To identify antenally-active components, coupled gas chromatography/ electroantennographic detection (GC-EAD) analysis was carried out on a Hewlett-Packard 7890B Series II gas chromatograph (Agilent Technologies, Palo Alto, California, USA) fitted with an HP-5MS capillary column (dimensions and oven conditions were the same as that of the GC/MS described earlier). High purity nitrogen was used as a carrier gas at a flow rate of 1 mL/min and injection was done in a splitless mode at 250 °C with a split valve delay of 1 min. With the aid of a fused silica outlet splitter (Alltech Associates Inc. Deerfield, IL), the column effluent was split into a 1:1 ratio for simultaneous detection by electroantennographic detector (EAD) and flame ionization detector (FID). Reference and recording electrodes made of silver wires (1.5 mm internal diameter) were immersed in glass capillaries electrodes filled with Ringer solution (0.24 g KCl, 1.36 g KH_2_PO_4_, 1.22 g MgCl_2_, 0.08 g CaCl_2_, 4.8 ml KOH, 35.08 g C_6_H_12_O_6_, and 0.35 g NaCl dissolved in 0.5 L of distilled water). The base of the excised antennae of gravid females of *P. absoluta* (2–3 days old) and *S. japonicus* (1–2 days old) and both sexes of *N. tenuis* adults (2–7 days old) were connected to the reference electrode and the tip of the antennae were connected to the recording electrode. Both electrodes were connected to an AC/DC amplifier in DC mode (Syntech, Kirchzarten, Germany). For FID and EAD signals detection, INR-II probe (Syntech, Hilversum, The Netherlands) and IDAC-2 data acquisition controller captured and processed the signals, and later analyzed them using GC-EAD 2000 (Syntech, Hilversum, The Netherlands) software on a computer. In each run, three microlitres (3 µL) of volatiles were analyzed and replicated three times using a fresh antenna. Compounds that elicited consistent EAD responses (at least three) were considered as EAD-active components. GC-MS was used to identify the EAD-active compounds.

### Chemicals

The synthetic standards including hexanal, (*E*)-2-hexenal, (*Z*)-3-hexenol, (*Z*)-3-hexenyl acetate, α-pinene, camphene, β-pinene, β-myrcene, α-terpinene, γ-terpinene, methyl salicylate (MeSA), and dichloromethane (DCM) were purchased from Sigma Aldrich (USA), whereas δ-2-carene, α-phellandrene, δ-3-carene, *p*-cymene, β-phellandrene, α-cedrene, δ-elemene, (*E*)-caryophyllene, α-humulene were purchased from Merck (France) and were used to confirm the identity of the compounds. The chemical purity of the synthetic standards except for α-phellandrene (85%), ranged between 90 and 99%.

### Bioassays with Synthetic Standards

Behavioral responses of gravid females of *P. absoluta*, *S. japonicus*, and both sexes of *N. tenuis* adults were tested using synthetic standards of the EAD-active components identified from the intact and *N. tenuis*-infested tomato plants in a dual choice Y-tube olfactometer as described earlier. The responses of *P. absoluta*, *S. japonicus*, and *N. tenuis* to each conspecific EAD-active component were tested individually at the natural, half-, and double-the natural concentrations detected in the plants (Tables 2 and 3) against the solvent control (DCM). The responses of each insect were also tested to blends 1, 2, and 3, respectively at the natural concentrations detected in the plants against the solvent control (DCM) (Tables 2 and 3).

**Table 3:** Electroantennographic (EAD)-detected components from intact and *Nesidiocoris tenuis-*infested plant synthetic standards tested

Insect	Electroantennographic (EAD)-detected components	Blend
*Phthorimaea absoluta*	hexanal, α-pinene, β-myrcene, δ-2-carene, α-phellandrene, β-phellandrene, (*Z*)-3-hexenyl butanoate, and δ-elemene	blend 1
*Stenomesius japonicus*	(*E*)-2-hexenal, (*Z*)-3 hexenol, δ-2-carene, β-phellandrene, γ-terpinene, (*Z*)-3-hexenyl butanoate, methyl salicylate, and (*E*)-caryophyllene	blend 2
*Nesidiocoris tenuis*	(*E*)-2-hexenal, δ-2-carene, α-terpinene, β-phellandrene, γ-terpinene, δ-elemene, and (*E*)-caryophyllene	blend 3

The synthetic blends were prepared from a stock solution containing 1 mg/ml of each compound in dichloromethane (DCM). The working solution or concentration of each compound used in the behavioral assays was then formulated from the stock solution separately. Equal volumes of the working solution of each compound were then pipetted individually and combined to obtain the various blends. This procedure was followed to formulate all the blends used in the study. In each assay, 10 µL aliquot of the test solution were loaded onto a filter paper (2 × 2 cm). The impregnated filter paper was air-dried at room temperature for 30 s before being used for the assay and was changed for every insect tested.

### Statistical Analyses

The frequency count from the Y-tube olfactometer bioassays was analyzed using Chi-square (χ^2^) goodness-of-fit analysis. The number of insects responding to each treatment in each experiment was expressed as a percentage using the formula [(n/N) × 100], where n is the number of responding insects to a given treatment, while N is the total number of insects responding. Non-responding insects were not included in the analysis.

A Shapiro-Wilk’s test was used to check for normality in the data from volatile emissions of the intact and *N. tenuis*-infested tomato plants. Further, since the data was not normally distributed, a Kruskal-Wallis test was performed followed by post-hoc comparisons using Dunn’s test with Bonferroni’s adjustment. Student T-tests were used to analyze where a compound was detected in only two treatment groups.

A multidimensional clustering analysis was done to visualize the similarity of the odor profiles of the intact and *N. tenuis*-infested tomato plants using a sparse partial least square discriminant analysis (sPLS-DA) using the mixOmics package (Lê Cao et al. 2011). All data were analyzed using R software (version 4.3.1) and the R Studio graphical user interface.

## Results

### Olfactometer Assays

#### Responses of *Phthorimaea absoluta* to Volatiles from Intact and *N. tenuis* Infested Tomato Plants

**Fig. 1:**
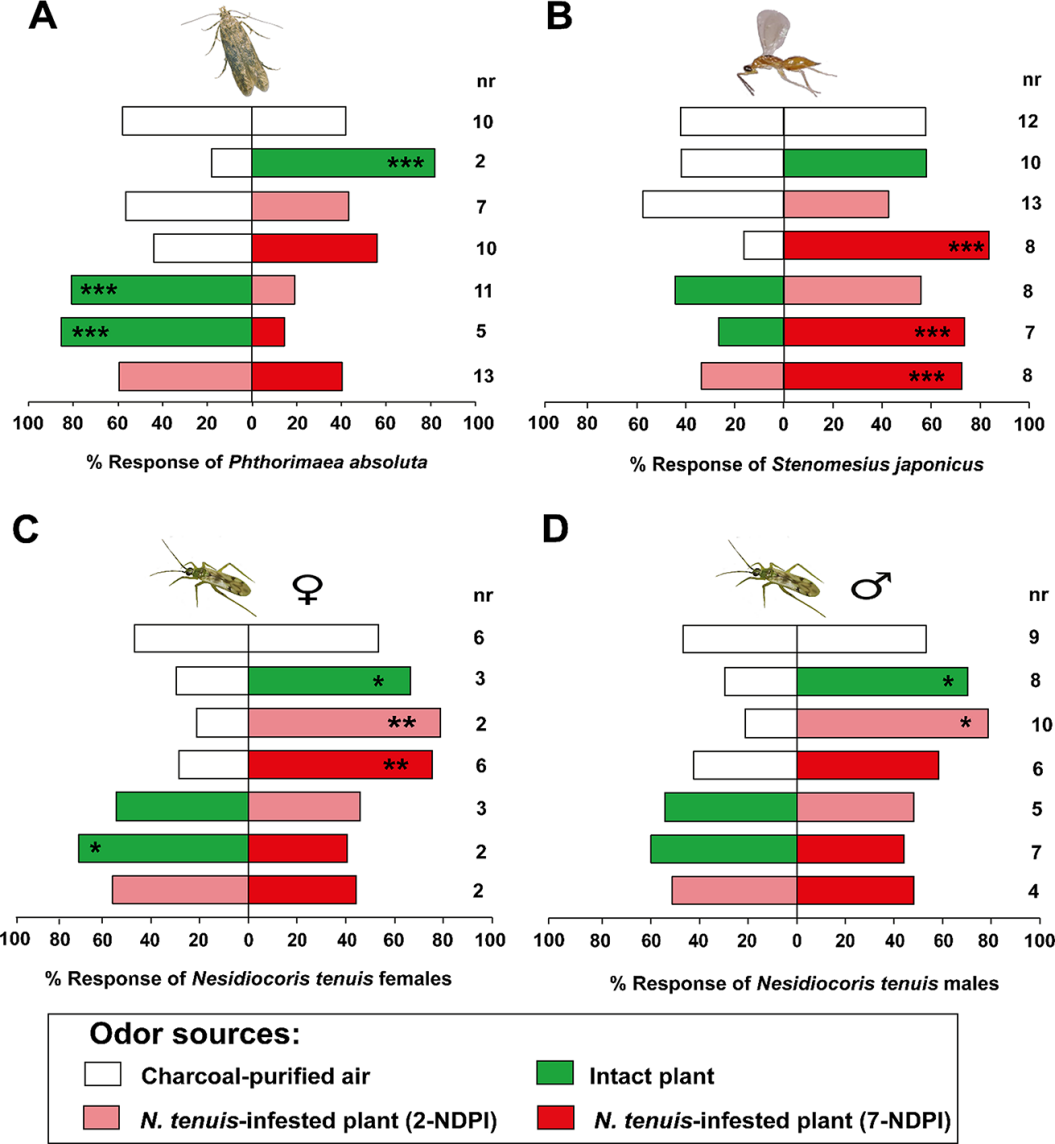
Behavioral responses of (**A**) *Phthorimaea absoluta* females, (**B**) *Stenomesius japonicus* females, and *Nesidiocoris tenuis* adult (**C**) males (♂) and (**D**) females (♀) to intact and *N. tenuis*-infested tomato plants. Asterisks (*) indicate significant differences (Chi-square test: *P < 0.05, **P < 0.01, ***P < 0.001). Non-responding insects (nr) were excluded from the analysis

Gravid females of *P. absoluta* showed no significant responses to the two arms of the olfactometer in control tests using the empty roasting bags alone (χ^2^ = 0.98, df = 1, *P* > 0.05) (Fig. 1A). Females of *P. absoluta* were significantly attracted to odours from intact (χ^2^ = 21.12, df = 1, *P* < 0.001) tomato plants relative to the air controls (Fig. 1A). Females of the moth did not show any preference for odours from *N. tenuis*-infested tomato plants at 2-NDPI (χ^2^ = 0.68, df = 1, *P* > 0.05) and 7-NDPI (χ^2^ = 0.50, df = 1, *P* > 0.05) relative to the air controls (Fig. 1A). When females of *P. absoluta* were presented with odours from intact and *N. tenuis*-infested plants, they significantly chose intact tomato plant odors relative to odours from *N. tenuis*-infested plants at 2-NDPI (χ^2^ = 16.68, df = 1, *P* < 0.001) and 7-NDPI (χ^2^ = 26.26, df = 1, *P* < 0.001), respectively (Fig. 1A). Interestingly, females of *P. absoluta* did not make any preference (χ^2^ = 1.36, df = 1, *P* > 0.05) when presented with odours from only *N. tenuis*-infested (2-NDPI and 7-NDPI) tomato plants in a pairwise comparison (Fig. 1A).

#### Responses of *Stenomesius japonicus* to Volatiles from Intact and *N. tenuis* Infested Tomato Plants

Control tests with empty roasting bags alone showed no significant responses from gravid females of *S. japonicus* (χ^2^ = 0.80, df = 1, *P* > 0.05) to the two arms of the olfactometer (Fig. 1B). In pairwise assays, gravid females of *S. japonicus* showed no preference for odours from intact tomato plants (χ^2^ = 0.98, df = 1, *P* > 0.05) and *N. tenuis* 2-NDPI plants (χ^2^ = 0.77, df = 1, *P* > 0.05) relative to the solvent control (Fig. 1B). However, *N. tenuis* 7-NDPI plants (χ^2^ = 20.89, df = 1, *P* < 0.001) elicited significant attractive responses in females of the parasitoid relative to the air controls (Fig. 1B). Similarly, when *S. japonicus* females were presented with odours from intact and *N. tenuis*-infested tomato plants, females of the parasitoid were significantly attracted to odours from *N. tenuis* 7-NDPI plants (χ^2^ = 10.87, df = 1, *P* < 0.001), but showed no preference for odours from *N. tenuis* 2-NDPI plants (χ^2^ = 0.48, df = 1, *P* > 0.05) relative to the intact tomato plants (Fig. 1B). Moreover, when females of the parasitoid were presented with odours from only *N. tenuis*-infested tomato plants, *S. japonicus* females significantly preferred odours from 7-NDPI (χ^2^ = 19.22, df = 1, *P* < 0.001) plants relative to odours from 2-NDPI plants (Fig. 1B).

#### Responses of *Nesidiocoris tenuis* to Volatiles from Intact and Tomato with Their Conspecifics

Control tests with empty roasting bags alone showed no significant responses of female adults of *N. tenuis* (χ^2^ = 0.04, df = 1, *P* > 0.05) to the two arms of the olfactometer (Fig. 1C). Females of *N. tenuis* were significantly attracted to odours from intact tomato plants (χ^2^ = 5.33, df = 1, *P* < 0.05) and conspecific-infested plants at 2-NDPI (χ^2^ = 8.04, df = 1, *P* < 0.05) and 7-NDPI (χ^2^ = 7.04, df = 1, *P* < 0.01) relative to the air controls (Fig. 1C). When the predator females were presented with odours from intact tomato plants and conspecific infested tomato plants, they significantly preferred odours from intact tomato plants compared to conspecific-infested 7-NDPI (χ^2^ = 4.32, df = 1, *P* < 0.05) plants, but did not show preference for odours from intact plants relative to conspecific-infested 2-NDPI tomato plants (χ^2^ = 0, df = 1, *P* > 0.05) (Fig. 1C). Interestingly, *N. tenuis* females did not make any choice between odours from only conspecific 2-NDPI and 7-NDPI tomato plants when compared to each other (χ^2^ = 0.04, df = 1, *P* > 0.05) (Fig. 1C).

Males of *N. tenuis* in a control test with empty roasting bags also made no significant choice between the two arms of the olfactometer (χ^2^ = 0.19, df = 1, *P* > 0.05) (Fig. 1D). The males of the predator were significantly attracted to odours from intact tomato plants (χ^2^ = 5.5, df = 1, *P* < 0.05) and conspecific-infested plants at 2-NDPI (χ^2^ = 4.05, df = 1, *P* < 0.05) relative to the control, but did not choose between odours from 7-NDPI plants and the control (χ^2^ = 1.04, df = 1, *P* > 0.05) (Fig. 1D). When presented with odours from intact tomato plants and conspecific-infested plants, *N. tenuis* males did not make a significant choice between odours from 2-NDPI plants (χ^2^ = 0, df = 1, *P* > 0.05) and 7-NDPI plants (χ^2^ = 0, df = 1, *P* > 0.05) relative to the intact plants (Fig. 1D). Likewise, when presented with odours from only conspecific-infested plants, males of the mirid did not choose between odours from 2-NDPI plants relative to the 7-NDPI plants (χ^2^ = 0, df = 1, *P* > 0.05) (Fig. 1D).

### Analysis of Volatiles

A total of 26 volatile organic compounds (VOCs) were detected in the odours from intact and *N. tenuis*-infested tomato plants belonging to four chemical classes: aldehydes, alcohols, terpenes, and esters (Table 2). There were 14 monoterpenes, 6 sesquiterpenes, 3 esters, 2 aldehydes, and 1 alcohol which differed quantitatively and qualitatively between the VOCs of intact and *N. tenuis*-infested tomato plants (Table 2). Herbivory of *N. tenuis* induced the release of specific VOCs such as (*E*)-2-hexenal, (*Z*)-3-hexenol, hexanal, *o*-cymene, β-pinene, δ-2-carene, *p*-cymene, γ-terpinene, (*E*)-isolimonene, (*Z*)-3-hexenyl acetate, (*Z*)-3-hexenyl butanoate, methyl salicylate, α-guaiene, δ-elemene, (*E*)-caryophyllene, α-humulene and cadina-1,4-diene (Table 2).

### Multidimensional Clustering of Intact and *N. tenuis*-Infested Tomato Plants

**Fig. 2:**
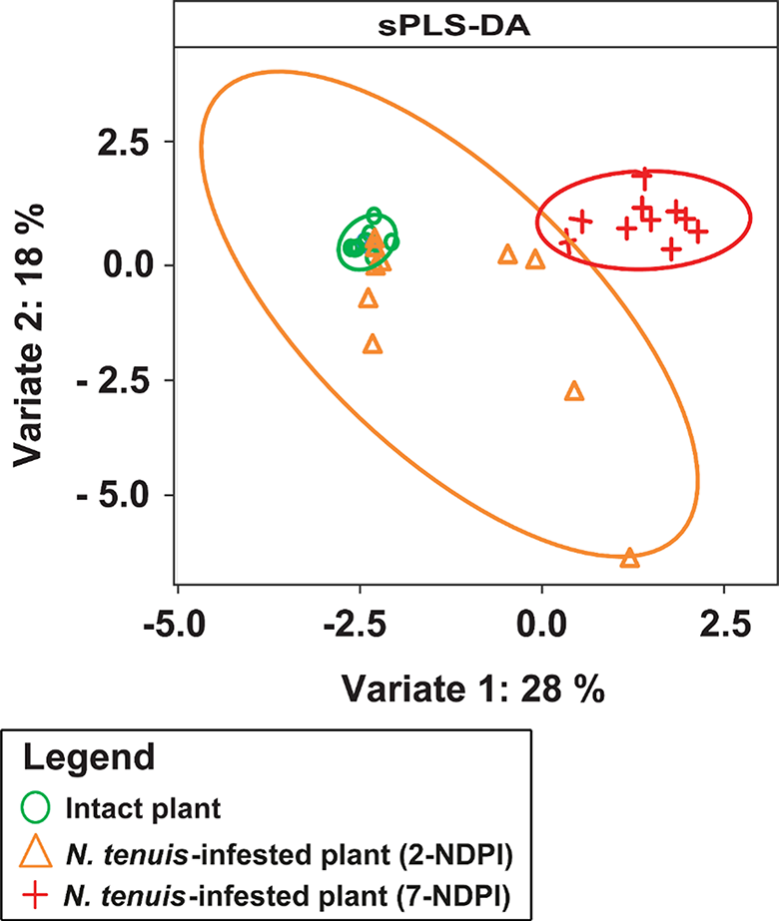
Sparse partial least square discriminant analysis (sPLS-DA) multidimensional scaling plot showing the distribution of intact and *N. tenuis*-infested tomato plant volatile profiles

Using the machine learning algorithm sparse partial least square discriminant analysis (sPLS-DA), the plants were grouped into clusters based on their volatile profiles (Fig. 2). *Nesidiocoris tenuis*-infested tomato plants clustered differently from the intact tomato plants. However, *N. tenuis*-infested 2-NDPI plants clustered closely with the intact plants whereas the 7-NDPI plants clustered completely differently from the 2-NDPI and intact plants (Fig. 2). The down-regulation of *p*-cymene in *N. tenuis*-infested 2-NDPI plants and the up-regulation of hexanal, *o*-cymene, β-pinene, δ-2-carene, γ-terpinene, δ-elemene, (*E*)-caryophyllene, and α-humulene in *N. tenuis*-infested 7-NDPI plants coupled with the induction of (*E*)-2-hexenal, (*Z*)-3-hexenyl acetate, (*E*)-isolimonene, (*Z*)-3-hexenyl butanoate, α-cedrene, methyl salicylate, α-guaiene, and cadina-1,4-diene in the *N. tenuis*-infested plants compared to the intact plants (Table 2) contributed to the differential clustering of the various plant groups. The total variations in the sPLS-DA (46%) were explained by the first two dimensions, with dimensions 1 and 2 accounting for 28% and 18%, respectively (Fig. 2).

### Electrophysiology

**Fig. 3:**
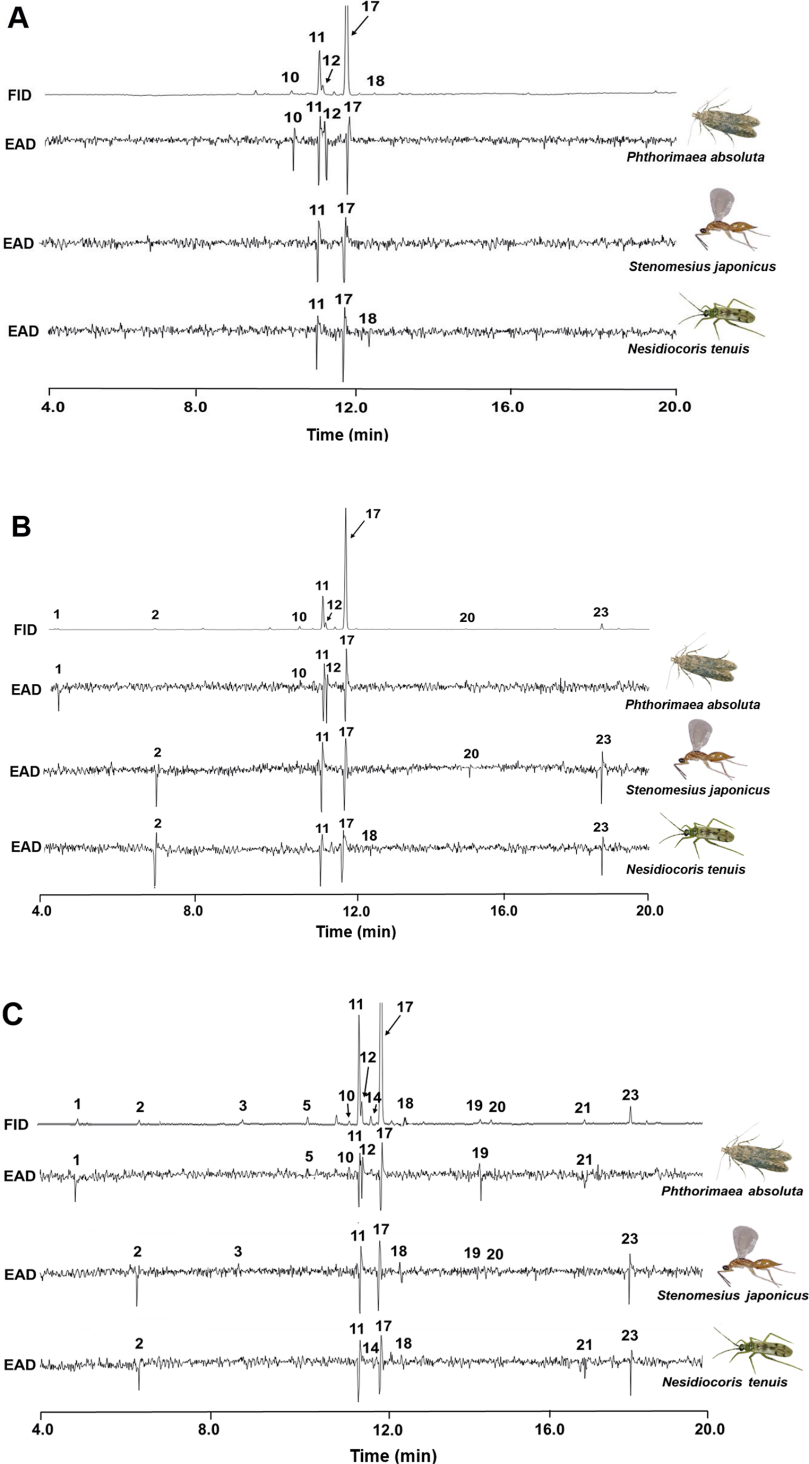
GC-EAD-active compounds from (**A**) intact tomato plant, (**B**) 2-days (2-NDPI)- and (**C**) 7-days (7-NDPI)- *Nesidiocoris tenuis*-induced tomato plant volatiles. The following EAD-active compounds were identified for *P. absoluta*: hexanal (1), α-pinene (5), β-myrcene (10), δ-2-carene (11), α-phellandrene (12), β-phellandrene (17), (*Z*)-3-hexenyl butanoate (19), and δ-elemene (21). *Stenomesius japonicus* detected: (*E*)-2-hexenal (2), (*Z*)-3-hexenol (3), δ-2-carene (11), β-phellandrene (17), γ-terpinene (18), (*Z*)-3-hexenyl butanoate (19), methyl salicylate (20), and (*E*)-caryophyllene (23). *Nesidiocoris tenuis* detected: (*E*)-2-hexenal (2), δ-2-carene (11), α-terpinene (14), β-phellandrene (17), γ-terpinene (18), δ-elemene (21), and (*E*)-caryophyllene (23)

GC-EAD analysis of intact and *N. tenuis*-infested tomato plant odors showed that the antennae of females of both *P. absoluta* and *S. japonicus* each detected eight EAD-active components, and the antennae of both sexes of *N. tenuis* adults detected seven EAD-active components which were identified by GC-MS. For *P. absoluta*, the identified components included β-myrcene (10), δ-2-carene (11), α-phellandrene (12), and β-phellandrene (17) from the intact tomato plant volatiles (Fig. 3A). An additional component was detected by the antennae of the moth identified as hexanal (1) from 2-NDPI plant volatiles (Fig. 3B). Additionally, in 7-NDPI plant volatiles, the antennae of *P. absoluta* further detected α-pinene (5), (*Z*)-3-hexenyl butanoate (19), and δ-elemene (21) (Fig. 3C; Table 2).

On the other hand, for *S. japonicus* the EAD-active components from the intact tomato plant volatiles were δ-2-carene and (11), β-phellandrene (17) (Fig. 3A) with further detection of the components (*E*)-2-hexenal (2), methyl salicylate (20), and (*E*)-caryophyllene (23) from 2-NDPI plants (Fig. 3B). In 7-NDPI plant volatiles, antennae of the parasitoid detected additional components identified as (*Z*)-3 hexenol (3), γ-terpinene (18), and (*Z*)-3-hexenyl butanoate (19) (Fig. 3C; Table 2).

For both sexes of *N. tenuis* adults, the EAD-active components were identified as δ-2-carene (11), β-phellandrene (17), and γ-terpinene (18) from the intact tomato plant volatiles (Fig. 3A) with additional components detected in 2-NDPI plant volatiles identified as (*E*)-2-hexenal (2) and (*E*)-caryophyllene (23) (Fig. 3B). In 7-NDPI plant volatiles, the mirid detected additional components α-terpinene (14) and δ-elemene (21) (Fig. 3C; Table 2).

The common EAD-active components detected by the antennae of *P. absoluta*, *S. japonicus*, and *N. tenuis* were δ-2-carene (11) and β-phellandrene (17). On the other hand, hexanal (1), α-pinene (5), β-myrcene (10), and α-phellandrene (12) were detected by the antennae of only *P. absoluta* females. Moreover, whereas (*Z*)-3 hexenol (3) and methyl salicylate (20) were specific to *S. japonicus*, α-terpinene (14) was specific to *N. tenuis* (Fig. 3; Table 2).

### Behavioral Responses to Synthetic Compounds

**Fig. 4:**
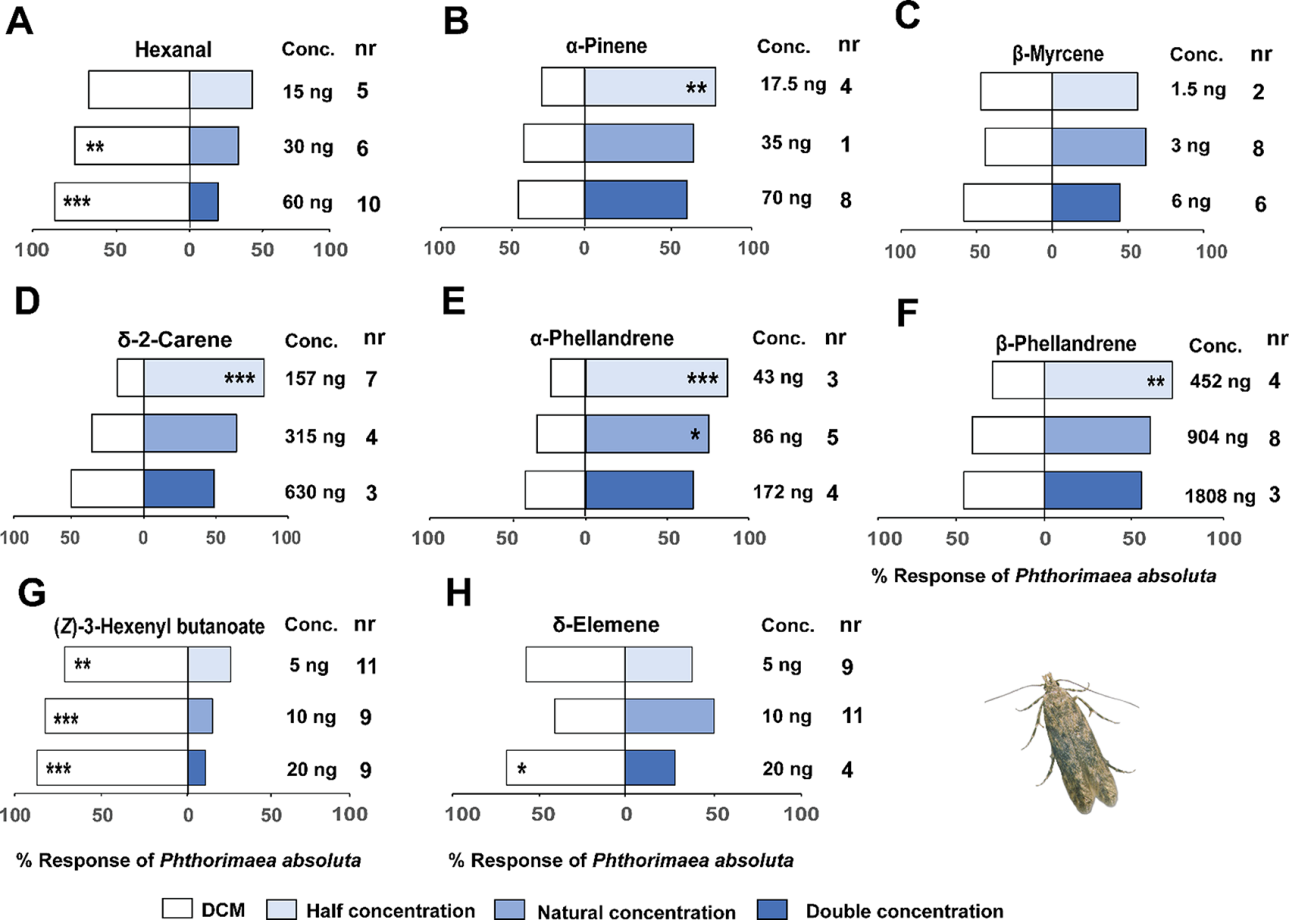
Behavioral response of *Phthorimaea absoluta* females to synthetic compounds in a Y-tube olfactometer. These compounds included: (**A**) hexanal (**B**) α-pinene, (**C**) β-myrcene, (**D**) δ-2-carene, (**E**) α-phellandrene, (**F**) β-phellandrene, (**G**) (*Z*)-3-hexenyl butanoate, and (**H**) δ-elemene. Asterisks (*) indicate significant differences (Chi-square test: *P < 0.05, **P < 0.01, ***P < 0.001). Non-responding insects (nr) were excluded from the analysis

Bioassays with the individual synthetic standards, hexanal at the natural concentration detected in *N. tenuis*-infested plants (30 ng/µL) (χ^2^ = 8.17, df = 1, *P* < 0.01) and double the natural concentration (60 ng/µL) (χ^2^ = 20.94, df = 1, *P* < 0.001) elicited avoidance behavior in *P. absoluta* females relative to the solvent controls (Fig. 4). Likewise (*Z*)-3-hexenyl butanoate at all concentrations tested including half (5 ng/µL) (χ^2^ = 8.82, df = 1, *P* < 0.01), natural (10 ng/µL) (χ^2^ = 20.90, df = 1, *P* < 0.001), and double (20 ng/µL) (χ^2^ = 28.31, df = 1, *P* < 0.001) the natural concentration were avoided by females of *P. absoluta* relative to the solvent controls (Fig. 4). Similarly, δ-elemene at double the natural concentration (20 ng/µL) (χ^2^ = 6.45, df = 1, *P* < 0.01) elicited avoidance behavior in females of *P. absoluta* relative to the solvent controls (Fig. 4). In contrast, at half the natural concentration, α-pinene (17.5 ng/µL) (χ^2^ = 9.45, df = 1, *P* < 0.01) and δ-2-carene (157 ng/µL) (χ^2^ = 19.32, df = 1, *P* < 0.001) elicited attractive responses in females of *P. absoluta* relative to the solvent controls (Fig. 4). Similarly, α-phellandrene at half the natural concentration (43 ng/µL) (χ^2^ = 13.75, df = 1, *P* < 0.001), natural concentration (86 ng/µL) (χ^2^ = 4.65, df = 1, *P* < 0.01) as well as β-phellandrene at half the natural concentration (452 ng/µL) (χ^2^ = 7.88, df = 1, *P* < 0.01) attracted females of *P. absoluta* relative to the solvent controls (Fig. 4).

**Fig. 5:**
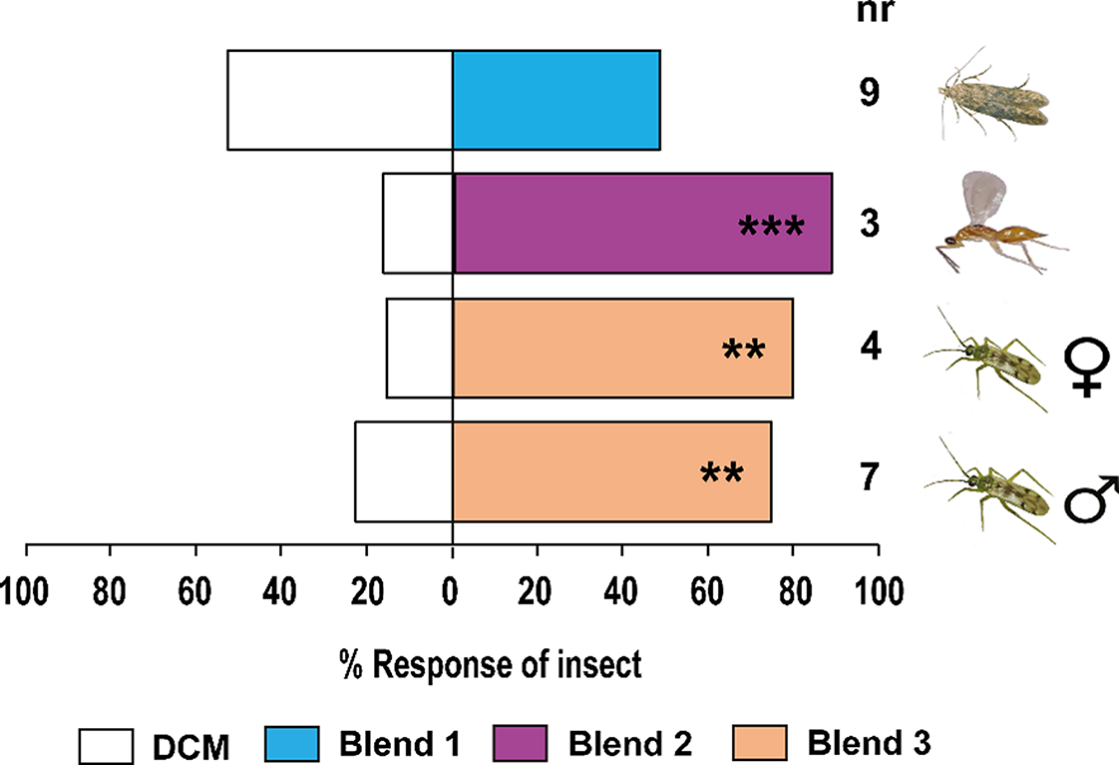
Behavioral responses of *Phthorimaea absoluta* females, *Stenomesius japonicus* females, and *Nesidiocoris tenuis* adult males (♂) and females (♀) to synthetic compounds in a Y-tube olfactometer. Blend 1 (eight-component blend) consists of *P. absoluta* females detected EAD-active components including hexanal, α-pinene, β-myrcene, δ-2-carene, α-phellandrene, β-phellandrene, (*Z*)-3-hexenyl butanoate, and δ-elemene. Blend 2 (eight-component blend) is composed of *S. japonicus* detected EAD- active components comprising (*E*)-2-hexenal, (*Z*)-3 hexenol, δ-2-carene, β-phellandrene, γ-terpinene, (*Z*)-3-hexenyl butanoate, methyl salicylate, and (*E*)-caryophyllene whereas blend 3 (seven-component blend) consists of EAD-active components detected by *N. tenuis* adults including (*E*)-2-hexenal, δ-2-carene, α-terpinene, β-phellandrene, γ-terpinene, δ-elemene, and (*E*)-caryophyllene. All the blends were formulated to simulate the natural concentrations detected in the plants (Table 2). Asterisks (*) indicate significant differences (Chi-square test: *P < 0.05, **P < 0.01, ***P < 0.001). Non-responding insects (nr) were excluded from the analysis

The blend of the eight EAD-active compounds formulated to simulate the amounts detected in *N. tenuis*-infested plant volatiles at the natural concentration (blend 1) did not elicit a significant behavioral response in *P. absoluta* females (χ^2^ = 0.88, df = 1, *P* > 0.05) relative to the solvent controls (Fig. 5).

**Fig. 6:**
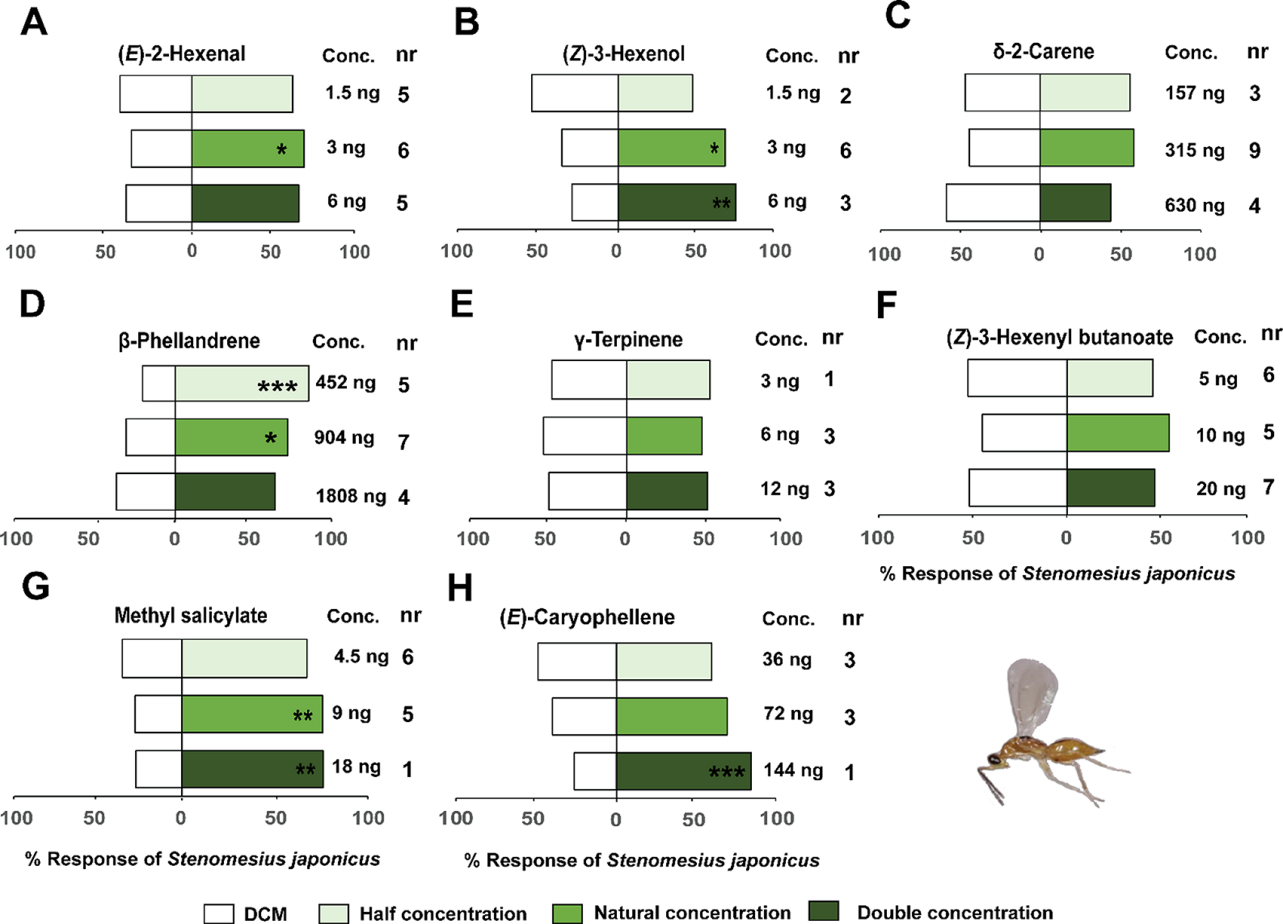
Behavioral response of *Stenomesius japonicus* females to synthetic compounds in a Y-tube olfactometer. These compounds included: (**A**) (*E*)-2-hexenal, (**B**) (*Z*)-3 hexenol, (**C**) δ-2-carene, (**D**) β-phellandrene, (**E**) γ-terpinene, (**F**) (*Z*)-3-hexenyl butanoate, (**G**) methyl salicylate, and (**H**) (*E*)-caryophyllene. Asterisks (*) indicate significant differences (Chi-square test: *P < 0.05, **P < 0.01, ***P < 0.001). Non-responding insects (nr) were excluded from the analysis

For the parasitoid, test with the individual EAD-active components from *N. tenuis*-induced volatiles at the natural concentrations for (*E*)-2-hexenal (3 ng/µL) (χ^2^ = 4.17, df = 1, *P* < 0.05), and (*Z*)-3-hexenol (3 ng/µL) (χ^2^ = 3.89, df = 1, *P* < 0.04), and double this concentration (6 ng/µL) (χ^2^ = 8.17, df = 1, *P* < 0.004) significantly attracted females of *S. japonicus* relative to the solvent controls (Fig. 6). Additionally, methyl salicylate at the natural concentration (9 ng/µL) (χ^2^ = 8.8, df = 1, *P* < 0.01) and double the natural concentration (18 ng/µL) (χ^2^ = 9.76, df = 1, *P* < 0.01) significantly attracted *S. japonicus* females relative to the solvent controls (Fig. 6). Moreover, β-phellandrene at half the natural concentration (452 ng/µL) (χ^2^ = 14.26, df = 1, *P* < 0.001) and the natural concentration (904 ng/µL) (χ^2^ = 3.69, df = 1, *P* = 0.04) as well as (*E*)-caryophyllene at double the natural concentration (144 ng/µL) (χ^2^ = 13.29, df = 1, *P* < 0.001) significantly attracted *S. japonicus* females relative to the solvent controls (Fig. 6).

Females of *S. japonicus* were significantly attracted to a blend of the eight EAD-active-components (blend 2) formulated to simulate the amounts detected in *N. tenuis*-infested plant volatiles at the natural concentration (χ^2^ = 21.88, df = 1, *P* < 0.001) relative to the solvent controls (Fig. 5).

**Fig. 7:**
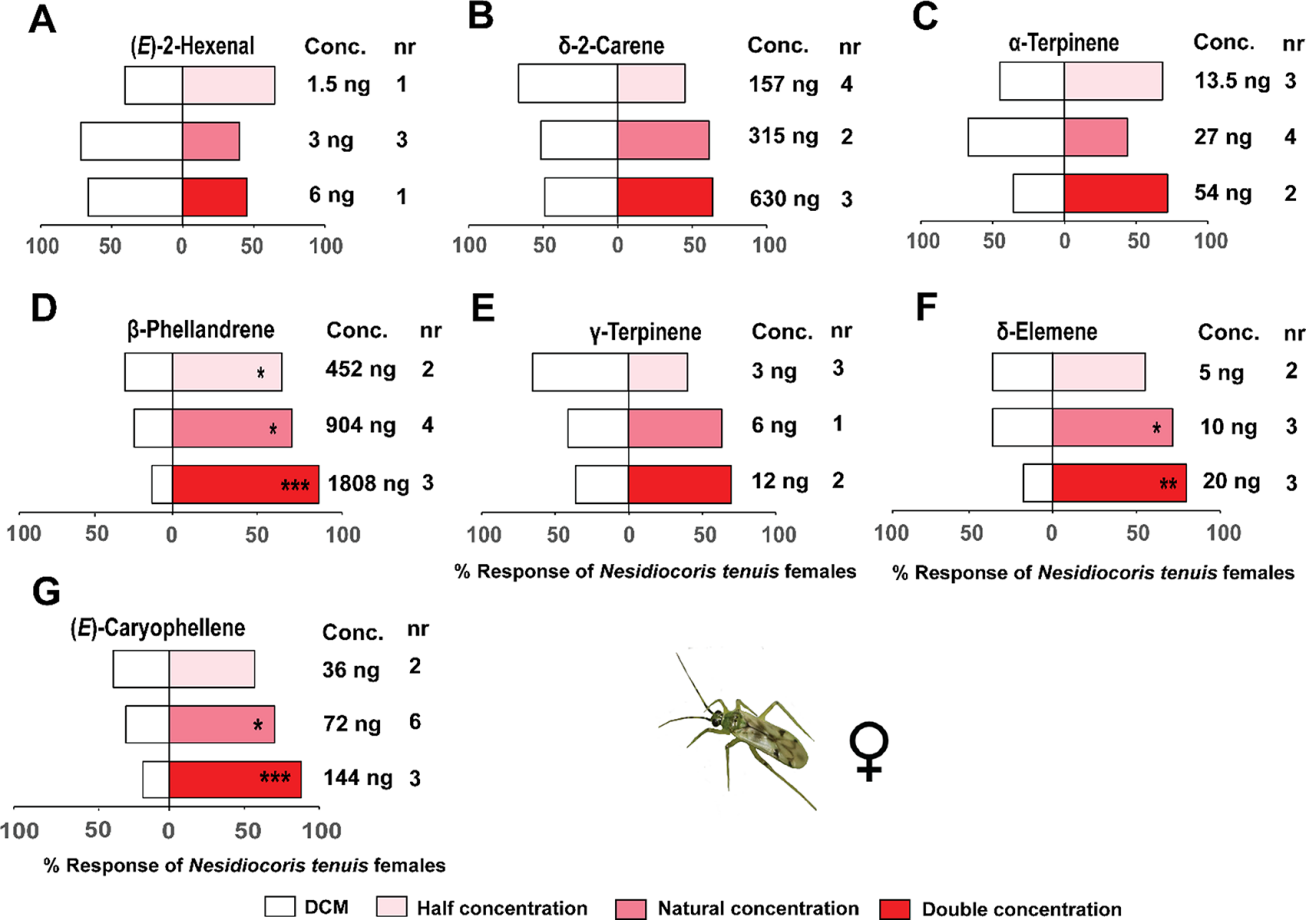
Behavioral response of *Nesidiocoris tenuis* adult females to synthetic compounds in a Y-tube olfactometer. These compounds included: (**A**) (*E*)-2-hexenal, (**B**) δ-2-carene, (**C**) α-terpinene, (**D**) β-phellandrene, (**E**) γ-terpinene, (**F**) δ-elemene, and (**G**) (*E*)-caryophyllene. Asterisks (*) indicate significant differences (Chi-square test: *P < 0.05, **P < 0.01, ***P < 0.001). Non-responding insects (nr) were excluded from the analysis

For the predator, tests with different concentrations of the individual components showed that *N. tenuis* females were attracted to β-phellandrene, at the natural (904 ng/µL) (χ^2^ = 5.04, df = 1, *P* < 0.05) and double the natural concentrations (1808 ng/µL) (χ^2^ = 12, df = 1, *P* < 0.001) relative to the solvent controls (Fig. 7). Likewise, relative to the solvent controls, *N. tenuis* females were significantly attracted to δ-elemene at half (5 ng/µL) (χ^2^ = 4.02, df = 1, *P* < 0.05) and double (20 ng/µL) (χ^2^ = 11.86, df = 1, *P* < 0.001) the natural concentrations as well as (*E*)-caryophyllene at double the natural concentration (144 ng/µL) (χ^2^ = 13.5, df = 1, *P* < 0.001) (Fig. 7).

**Fig. 8:**
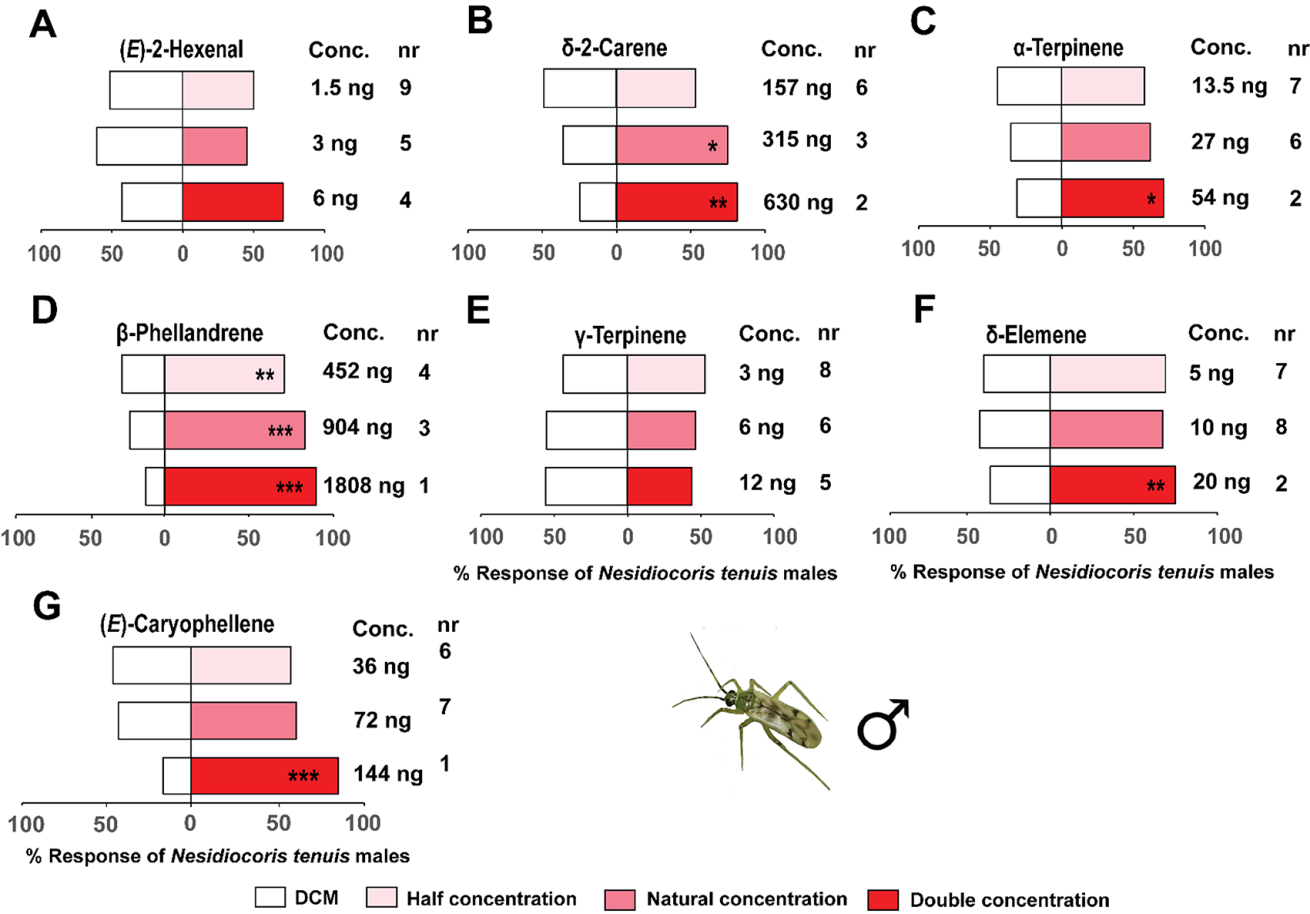
Behavioral response of *Nesidiocoris tenuis* adult males to synthetic compounds in a Y-tube olfactometer. These compounds included: (**A**) (*E*)-2-hexenal, (**B**) δ-2-carene, (**C**) α-terpinene, (**D**) β-phellandrene, (**E**) γ-terpinene, (**F**) δ-elemene, and (**G**) (*E*)-caryophyllene. Asterisks (*) indicate significant differences (Chi-square test: *P < 0.05, **P < 0.01, ***P < 0.001). Non-responding insects (nr) were excluded from the analysis

Similarly, males of the mirid predator were significantly attracted to β-phellandrene, at the natural (904 ng/µL) (χ^2^ = 12, df = 1, *P* < 0.001), half (χ^2^ = 8.65, df = 1, *P* < 0.01), and double the natural concentrations (1808 ng/µL) (χ^2^ = 13.79, df = 1, *P* < 0.001) relative to the solvent controls (Fig. 8). Adult *N. tenuis* males were significantly attracted to only double the natural concentrations of δ-elemene (20 ng/µL) (χ^2^ = 8.03, df = 1, *P* < 0.05), (*E*)-caryophyllene (144 ng/µL) (χ^2^ = 11.17, df = 1, *P* < 0.001), and α-terpinene (54 ng/µL) (χ^2^ = 6.04, df = 1, *P* < 0.05) relative to the solvent controls (Fig. 8). Additionally, males of *N. tenuis* were significantly attracted to δ-2-carene at the natural concentration (315 ng/µL) (χ^2^ = 5.33, df = 1, *P* < 0.05) and double the natural concentration (630 ng/µL) (χ^2^ = 8.04, df = 1, *P* < 0.05) relative to the solvent controls (Fig. 8).

Both adult sexes of the predator *N. tenuis* were significantly attracted to a blend of the seven conspecific EAD-active components (blend 3) formulated to simulate the natural concentrations in the conspecific-infested plant volatiles, females (χ^2^ = 8.65, df = 1, *P* < 0.05) and males (χ^2^ = 8.52, df = 1, *P* < 0.05) relative to the solvent controls (Fig. 5).

## Discussion

We investigated the responses of *P. absoluta*, its associated larval parasitoid *S. japonicus*, and the mirid predator *N. tenuis* to constitutive and herbivory- and oviposition-induced volatiles by *N. tenuis* on tomato plants. Our results indicate that both *P. absoluta* and *N. tenuis* are attracted to constitutive host plant volatiles, but the parasitoid *S. japonicus* is indifferent. The results further indicate that *P. absoluta* females avoid volatiles from *N. tenuis-*infested plants, but *S. japonicus* females and *N. tenuis* adults are attracted to the infested plant volatiles. These findings demonstrate the role *N. tenuis*-induced host plant defense volatiles play in the tritrophic interaction. We used GC-EAD and GC-MS analyses and behavioral assays to identify the olfactory-mediating chemicals.

The differential responses of the prey, predator, and parasitoid to the constitutive volatiles from the intact host tomato plants in this study can be explained by the fact that both *P. absoluta* and *N. tenuis* are phytophagous while the parasitoid *S. japonicus* is carnivorous. Gravid females of *P. absoluta* and both sexes of adult *N. tenuis* may be attracted to constitutive volatiles for suitable oviposition sites, conducive habitat selection, or the fitness benefits of their progeny which includes access to nutritious food sources and protection from their natural enemies. It is reasonable for *N. tenuis* to be attracted to constitutive tomato volatiles because previous studies have demonstrated that mirid predators prefer to colonize host plants to build up their populations before the arrival of pests (Silva et al. 2018; Messelink et al. 2015). On the other hand, the lack of interest by the parasitoid *S. japonicus* in constitutive volatiles may be associated with the absence of a host/prey which aligns with previous studies on multi-trophic interactions mediated by constitutive volatiles (Gontijo et al., 2019; Pérez-Hedo et al. 2018; Lins et al. 2014). For example, in a laboratory study, intact host tomato plant volatiles elicited attractive responses in the tomato herbivore *P. absoluta* females, two mirid predators associated with the pest *N. tenuis* and *Macrolophus pygmaeus*, but the parasitoid *Encarsia formosa* also associated with the pest did not show any significant interest in the constitutive volatiles (Pérez-Hedo et al. 2018). However, in a different study, intact host tomato plant volatiles attracted *P. absoluta* females, but *N. tenuis* adults did not show any interest in the constitutive plant volatiles in a laboratory study (Ayelo et al., 2021). These contrasting results may be associated with plant cultivar and developmental stage, and insect strains which would require additional research.

The results of our study also indicate that there were differential responses of the prey *P. absoluta* and the two natural enemies *S. japonicus* and *N. tenuis* to the predator-induced host plant volatiles. Females of *P. absoluta* may avoid *N. tenuis*-induced plant volatiles to ensure the survival of their progeny such as reducing or avoiding predation of their eggs and progeny. In the case of the parasitoid *S. japonicus*, female attractive responses to the predator-induced host plant volatiles may be associated with searching for a host. As such, herbivore-induced volatiles irrespective of the herbivore may provide the parasitoid with habitat location signals. Other cues such as visual and vibrational cues may contribute to close-range discrimination of the host by the parasitoid which would be interesting to investigate (Ayelo et al. 2022). Our findings are in line with previous studies on multi-trophic interactions of other insects associated with tomato plants (Ayelo et al., 2021; Sarmah et al. 2022; Pasquale et al. 2023). For example, in a greenhouse study, the introduction of *N. tenuis* as a biological control agent significantly reduced the population of *P. absoluta* and *B. tabaci* on tomato plants attributed to the induction of host plant defense volatiles that may have repelled the pests and attracted other natural enemies associated with the herbivores (Pérez-Hedo et al. 2015b). Likewise, the population of *F. occidentalis* and *B. tabaci*, pests of sweet pepper plants were reduced, and the parasitoid *Encarsia formosa* was attracted to infested plants (Bouagga et al. 2018). However, the volatiles mediating these interactions were not identified by Bouagga et al. (2018). In the present study, the attraction of *N. tenuis* to conspecific-induced plant volatiles is consistent with a previous study (Lins et al. 2014). Natural enemies are known to locate their host using herbivore-induced volatile signals (Fiaboe et al. 2023; Ayelo et al. 2022). Our results and these previous findings contribute to the existing literature indicating the important role plant volatiles play in the host selection behavior of phytophagous insects.

Our chemical analysis revealed qualitative and quantitative differences in the headspace volatiles of intact and *N. tenuis*-infested tomato plants. Terpenes and GLVs dominated the volatile profiles of the tomato plant which may contribute directly and indirectly to its defense against the two herbivores, the leafminer and predator. This assertion was confirmed by our multidimensional analysis of the chemical profiles of the various plants whereby volatiles of intact and *N. tenuis-*infested tomato plants clustered differently. Previous studies have demonstrated that herbivory of sap-sucking or piercing and sucking insects (such as *N. tenuis*) activate the salicylic acid defense pathway in host plants resulting in the induction of specific VOCs such as the alcohol (Z)-3-hexenol and the esters (Z)-3-hexenyl acetate, (Z)-3-hexenyl butanoate, and methyl salicylate (Lins et al. 2014; Pérez-Hedo et al. 2015b; Yang et al. 2021). Additionally, we found that hexanal, *o*-cymene, β-pinene, δ-2-carene, γ-terpinene, δ-elemene, (*E*)-caryophyllene, and α-humulene were upregulated, whereas *p*-cymene was down-regulated. Interestingly, previous studies had shown that *P. absoluta* herbivory on tomato also causes upregulation of specific induced volatiles, of which δ-2-carene, γ-terpinene, δ-elemene, (*E*)-caryophyllene, and α-humulene are common between the two herbivores *P. absoluta* and *N. tenuis* (Fiaboe et al. 2023; Ayelo et al. 2022). These results suggest that the host plant tomato defense against herbivory may be non-specific to the herbivore species. Although our analysis of herbivore-induced volatiles aligns with these previous results, it is possible that the 24 h volatile collection period might cause breakthroughs of some low molecular weight components from the adsorbent.

Our GC-EAD results indicated that terpenes from constitutive volatiles and both terpenes and GLVs induced by *N. tenuis* phytophagy elicited antennal responses in females of *P. absoluta* and *S. japonicus* and both adult sexes of the mirid predator. Our previous study (Adams et al. 2023) demonstrated that terpenes influenced the host selection behavior of *P. absoluta* and *N. tenuis*. As such, it is unsurprising that in the present study, *P. absoluta* females and *N. tenuis* adults detected terpenes present in the constitutive and predator-induced volatiles. Terpenes may serve as host-finding cues for females of *P. absoluta*, whereas when combined with GLVs in predator-induced volatiles they may serve as a warning signal to females of the presence of natural enemies. However, females of *S. japonicus* and both sexes of *N. tenuis* adults may detect terpenes from constitutive volatiles as candidate hosts or habitat selection cues. Additionally, both adults of *N. tenuis* and females of *S. japonicus* may detect terpenes and GLVs from predator-induced volatiles to avoid competition for food and space with other conspecifics. Antennal detection of the constitutive host plant volatiles β-myrcene, δ-2-carene, α-phellandrene, and β-phellandrene by the prey *P. absoluta* females is consistent with previous findings (Fiaboe et al. 2023; Anastasaki et al. 2018). Additionally, antennal detection of the specific predator-induced VOCs hexanal, α-pinene, β-myrcene, δ-2-carene, α-phellandrene, β-phellandrene, δ-elemene, and (*Z*)-3-hexenyl butanoate by *P. absoluta* females has been reported in previous studies (Anastasaki et al. 2018; Fiaboe et al. 2023; Miano et al. 2022). It is worth noting that the antennae of *P. absoluta, S. japonicus*, and *N. tenuis* all detected the common components δ-2-carene and β-phellandrene, whereas hexanal, α-pinene, β-myrcene, α-phellandrene were specific to *P. absoluta* antennae, and (*Z*)-3-hexenol, methyl salicylate to *S. japonicus*, and α-terpinene to *N. tenuis*. These results indicate that the antennae of the three insect species are broadly tuned to detect GLVs and terpenes, and that blends of these compounds and those specific to each of them could serve as candidate attractants/repellents in the tritrophic interaction.

In behavioral assays, the fact that various blends composed of terpenes and GLVs from the constitutive and the predator-induced volatiles elicited significant differential responses in *P. absoluta*, *S. japonicus*, and *N. tenuis* suggest that the amounts and ratios of specific compounds may contribute to the overall quality of signals detected by herbivores. The prey *P. absoluta* females were attracted to the monoterpenes α-pinene, δ-2-carene, α-phellandrene, and β-phellandrene when tested individually, but were indifferent to the eight-component blend containing them. This suggests that the GLVs (*Z*)-3-hexenyl butanoate and hexanal and the sesquiterpene δ-elemene may have masked the attractiveness of the blend (Adams et al. 2023). It could also be the case that other important background components that enhance the attraction of *P. absoluta* were missing in the blends, however, further research comparing crude extract from host plants with synthetic blends is needed to confirm this possibility. Previous studies demonstrated that monoterpenes such as α-pinene, δ-2-carene, α-phellandrene, and β-phellandrene attracted *P. absoluta* females (Caparros Megido et al. 2014; Proffit et al. 2011; Subramani et al. 2021). Similarly, Ayelo et al. (2021) found that a five-component monoterpene blend (α-pinene, α-phellandrene, δ-3-carene, β-phellandrene, and β-ocimene) attracted the mirid predator *N. tenuis.* Additionally, in a previous study, *P. absoluta*- and *B. tabaci*-infested plants were found to be attractive to three related mirid predators, *Engytatus varians*, *Macrolophus basicornis*, and *Campyloneuropsis infumatus*, which was attributed to the elevated levels of the compounds β-phellandrene and δ-elemene because of herbivory (Silva et al. 2018). The fact that generalist mirid predators exploit quantitative and qualitative differences in volatile signals for habitat and host location may explain the differential attraction of *N. tenuis* adult males and females to specific terpenes (δ-2-carene, α-terpinene, β-phellandrene, δ-elemene and (*E*)-caryophyllene) (Lins et al. 2014; Pérez-Hedo et al. 2018), also found in the current study. Contrastingly, the GLV (*Z*)-3-hexenyl butanoate elicits a repellent response in *P. absoluta* females and *N. tenuis* adults (Fiaboe et al. 2023; Ayelo et al., 2021). Likewise, a previous study (Wang et al. 2020), reported the repellent role of hexanal in the behavior of the dark black chafer, *Holotrichia parallela* adults, a major soil insect pest of several plants.

In summary, we have demonstrated that in the tomato-*P. absoluta*- *S. japonicus/N. tenuis* tritrophic interaction, terpenes from constitutive host tomato plant volatiles serve as kairomones. Whereas a combination of terpenes and GLVs from *N. tenuis*-infested tomato plants serve as allomones for *P. absoluta*. In contrast, GLVs from *N. tenuis*-induced host plant volatiles and a combination of terpenes and GLVs from both constitutive and *N. tenuis*-induced host plant volatiles serve as kairomones for *S. japonicus* and *N. tenuis*, respectively. Our findings lay the foundation for the potential integration of terpenes and GLVs in IPM strategies for the sustainable management of *P. absoluta*.

## Data Availability

The data generated from this study are available from the corresponding authors upon request.
